# Early experience with open heart surgery in a pioneer private hospital in West Africa: the Biket medical centre experience

**DOI:** 10.11604/pamj.2017.28.59.13188

**Published:** 2017-09-21

**Authors:** Uvie Ufuoma Onakpoya, Adebisi David Adenle, Anthony Taiwo Adenekan

**Affiliations:** 1 Department of Surgery, Obafemi Awolowo University, Ile-Ife, Nigeria; 2 Biket Medical Centre Osogbo, Osun Nigeria; 3 Department of Anaesthesia, Obafemi Awolowo University, Ile- Ife, Nigeria

**Keywords:** Open heart surgery, Nigeria, private hospital, pioneer cardiac surgery

## Abstract

**Introduction:**

More than forty years after the first open heart surgery in Nigeria, all open heart surgeries were carried out in government-owned hospitals before the introduction of such surgeries in 2013 at Biket Medical Centre, a privately owned hospital in Osogbo, South-western Nigeria. The aim of this paper is to review our initial experience with open heart surgery in this private hospital.

**Methods:**

All patients who underwent open heart surgery between August 2013 and January 2014 were included in this prospective study. The medical records of the patients were examined and data on age, sex, diagnosis, type of surgery, cardiopulmonary bypass details, complications and length of hospital stay were extracted and the data was analysed using SPSS version 16.

**Results:**

Eighteen patients comprising of 12 males and 6 females with ages ranging between 8 months and 52 years (mean= of 15.7 +/- 15 years) were studied. Pericardial patch closure of isolated ventricular septal defect was done in 7 patients (38.9%) while total correction of isolated tetralogy of Fallot was carried out in 5 patients (27.8%). Two patients had mitral valve repair for rheumatic mitral regurgitation. Sixty day mortality was 0%.

**Conclusion:**

Safe conduct of open heart surgery in the private hospital setting is feasible in Nigeria. It may be our only guarantee of hitch free and sustainable cardiac surgery.

## Introduction

More than forty years after the first open heart surgery was performed in Nigeria on the 1^st^ of February 1974 [1], there still does not exist any hospital in the country performing routine daily open heart surgeries. Patients with surgically correctable lesions often have to go outside Nigeria for their procedures or wait long periods because most hospitals currently offering open heart surgery services are government-owned and frequently have to pool patients for foreign-assisted missions. Also, many cardiac surgery missions do have a very strong adult heart surgery arm and only an infrequent congenital programme. Several factors culminating in the relative non-performance of the government hospitals have been previously outlined by various authors [2, 3]. In many countries in North and South Africa, most cardiac surgeries are performed in private hospitals that do not have the bureaucratic bottlenecks seen in publicly administered hospitals. Open heart surgery (OHS) is a high skill and technology service performed by a wide array of trained personnel. The financial outlay and setting for this has previously only been fulfilled by government-funded institutions in Nigeria. The availability of personnel was also very limited in Nigeria until recently when due to the surgical postgraduate residency training programme and interest of trainees in acquiring skills in high volume centres outside Nigeria, surgical competencies have improved [4, 5].

Also there has been a paradigm shift from the training of the cardiothoracic surgeon to the training of cardiac surgical teams [6] to include other members (anaesthetists, cardiologists, perfusionists, peri-operative and intensive care nurses, respiratory physiotherapists etc). This has enabled the number of public institutions that have carried out OHS to increase from one or two centres 10 years ago to more than seven presently. The Biket medical centre model constitutes local team members working in conjunction with two foreign teams to carry out surgeries together, with increasing responsibilities allocated to the local OHS team to enable them to be fully independent in a short time. This has entailed working visits to the partnering institutions to understudy and work with their teams especially after the second mission in January 2014. The largely unmet need in congenital heart surgery is one of the motivations in BMC's partnerships with the two congenital heart teams. To the best of our knowledge, there did not exist in the West African subregion, a private hospital that performed open heart surgery prior to our pioneering efforts. The Biket Medical Centre (BMC), in Osogbo, South-west Nigeria was the first privately owned hospital to perform open heart surgery in West Africa on the 23^rd^ of August 2013 and the aim of this paper is to review our initial 16-month experience of open heart surgeries at this facility.

## Methods

**Setting**: The BMC is a 48-bedded multispecialty hospital with a three-bed intensive care unit and a three-bed high dependency unit. It has 4 echocardiography machines with two trans-oesophageal probes (one adult and one paediatric), a Sarns 8000^TM^ cardiopulmonary bypass machine and heat exchanger, a cardiac catheterization laboratory, mobile x-ray facility and relevant consumables.

**Personnel**: There is one cardiologist, a cardiac surgeon, two cardiac anaesthesiologists, one intensivist, two cardiopulmonary perfusionists and peri-operative and ward nurses which comprise the local team. The BMC has strategic partnerships with two foreign hospitals (Sheba Medical Centre, Israel and Krishna Institute of Medical Sciences Hospital, India) that visit with their complement of staff during cardiac surgeries. This partnership helps with further training of the local team and confidence boosting of the population. This however necessitates the need to pool patients together so that the local and foreign teams can work together at mutually agreed times. We have had three cardiac missions within the period under review and this report includes all patients who underwent open heart surgeries.

**Statistical methods**: Records of all patients who underwent open heart surgery using a median sternotomy and cardiopulmonary bypass were prospectively collected to include the patient's age, sex, diagnosis, type of surgery, cardiopulmonary bypass details, post-operative complications and length of hospital stay and the data was analysed using SPSS version 16. Continuous variables were summarized using means and standard deviations or medians and inter-quartile ranges for highly skewed variables. Discrete variables were summarized as counts and percentages and P-values less than 0.05 were considered statistically significant.

## Results

A total of eighteen (18) patients were operated upon in the 16-month period. The patients had a median age of 5.25 years (age range=8 months-52 years) with 9 patients (50.0%) aged less than 5 years, (Table 1). There were 12 males (66.7%) and 6 females (33.3%) with a male: female ratio of 2:1 (Table 1). Most patients (77.7%; n = 14 patients) weighed ≤ 30kg (Table 1) and the patients in all had a mean weight of 24.8 +/-20.9kg (range = 6-70kg) and a body surface area of 0.86 +/-0.52m^2^. Most of the patients had mild clinical cardiac decompensation with 9 patients (50.0%) in New York Heart Association (NYHA) class 2 while 7 patients (38.9%) were in NYHA class 3 and only two patients (11.1%), were in NYHA class 4. Sixteen patients (88.9%) had congenital heart disease mainly of ventricular septal defect (Table 1). Glutaraldehyde-treated (0.6%) pericardial patch closure of isolated ventricular septal defect (VSD) was done in 7 patients (38.8%) while total correction of tetralogy of Fallot (TOF) was carried out in 5 patients (27.8%) while one child with combined TOF and single atrium had total correction and atrial septation using a large glutaraldehyde-treated autologous pericardial patch (Table 2).

**Table 1:** Patient characteristics

Patient characteristics	Frequency	%
**Sex**		
Male	12	66.7
Female	6	33.3
**Age (Years)**		
<5	9	50.0
5-20	6	33.3
>20	3	16.7
Median age: 5.25 years (Range= 8 months-52years)		
**Weight (Kg)**		
< 10	7	38.9
10- 30	7	38.9
>30	4	22.2
Mean weight: 24.8 +/- 20.9 (Range= 6- 70kg)		
**Body Surface Area (m^2^)**		
<0.5m^2^	7	38.9
0.5- 1.0m^2^	5	27.7
1.0- 1.5m^2^	3	16.7
> 1.5m^2^	3	16.7
Mean Body Surface Area: 0.86 +/- 0.52m^2^		
**New York Heart Association class**		
2	9	50.0
3	7	38.9
4	2	11.1
**Diagnosis**		
**Congenital heart disease**	16	83.3
Isolated VSD	7	(38.9%)
Tetralogy of Fallot	5	(27.8%)
DORV + sub- aortic VSD + SAM	1	(5.6%)
TOF + Single atrium	1	(5.6%)
VSD + Infundibular stenosis	1	(5.6%)
VSD + Patent ductus arteriosus	1	(5.6%)
**Acquired heart disease**	2	16.7
Severe rheumatic mitral regurgitation	2	16.7

VSD: ventricular septal defect; DORV-Double outlet right ventricle; SAM-Sub-aortic membrane; TOF: tetralogy of fallot

**Table 2:** Preoperative data

Surgery performed	Frequency	%
VSD Closure	9	50.0
Total correction of TOF	5	27.7
Mitral valve repair	2	11.1
Total correction of TOF + Atrial septation	1	5.6
Tunnelled pericardial patch +Resection of sub aortic membrane and hypertrophied sub- pulmonic muscle bands	1	5.6
**Cardiopulmonary BypassTime** 145.9 +/- 68.2 minutes (Range= 42- 270 minutes)		
**Aortic Cross-Clamp time** 99.3 +/- 47.8 minutes (Range= 24- 190 minutes)		
**Complications**		
Atelectasis	3	16.6
Bleeding	1	5.6
Transcient low cardiac output	1	5.6

One child (5.6%) with double outlet right ventricle, subpulmonic hypertrophied muscle bands, sub aortic VSD and a sub aortic membrane was managed by tunnelled pericardial patch closure, resection of both hypertrophied muscle bands and the sub aortic membrane. The two females (11.1%) with rheumatic mitral regurgitation aged 15 and 31 years had mitral valve repair consisting of chordal shortening and annuloplasty ring implantation (Table 2). All patients underwent surgery utilising aortic, bicaval cannulation with moderate hypothermia (28-30°C) and antegrade cold blood cardioplegia. The mean cardiopulmonary bypass time was 145.9 +/-68.2 minutes (Range = 42-270 minutes) and the aortic cross-clamp time was 99.3 +/-47.8 minutes (Range= 24-190 minutes). The cardiopulmonary bypass time for patients with tetralogy of Fallot was 187.7 +/-98.8 minutes compared with 124.2 +/-67.8 minutes for non-cyanotic heart diseases (p = 0.234). Post-operative mechanical ventilation was for 7.6 +/-3.7 hours while the post-operative intensive care unit stay was 45.9 +/-34.5 hours (Table 2). Total post-operative blood loss was 499.5 +/-444.2 mls (range= 110-1570mls) necessitating reoperation for bleeding in one patient (5.6%) who was a 20-year old man with tetralogy of Fallot. The post-operative hospital stay was 9.1 +/-2.4 days (Table 3) and the 60 day mortality was 0%. There was one late death at 13 months after surgery due to chronic renal failure in the child with double outlet right ventricle and sub-aortic membrane.

**Table 3:** Post-operative cost

Mechanical ventilation	7.6 +/- 3.7 hours
Intensive care unit stay	45.9 +/-34.5 hours
Blood loss	499.5 +/-444.2 mls
Total hospital stay	9.1 +/-2.4 days

## Discussion

The median age of patients (5.25 years) reflects our focus on congenital heart disease and would have been much lower but for one 52-year-old man with symptomatic ventricular septal defect and mild pulmonary hypertension. It is essential to operate children with congenital heart disease as quickly as possible and feasible before the onset of severe pulmonary hypertension and severe cardiac decompensation which are time dependent so, most of our patients were highly selected with few co-morbidities. This decision is well supported by other previous pioneers [7] in open heart surgery to achieve success thereby boosting the morale of the staff and the general population. Ventricular septal defect is the most common congenital cardiac defect seen in Nigeria [8–10] while tetralogy of Fallot is the commonest cyanotic heart disease [9]. This was our experience as 9 patients (50%) had ventricular septal defect either as an isolated condition or in association with patent ductus arteriosus and pulmonary stenosis. Severe rheumatic mitral regurgitation was seen in two female patients; a fifteen year old with chordal elongation of the primary chordae and relatively pliable leaflets and a 31-year-old lady with annular dilatation and chordal shortening of few secondary chordate. Both patients had mitral valve repair since they were in the reproductive age group; the 15-year-old, had chordal shortening and implantation of a 26mm semi-rigid mitral ring CG future^®^ 638R (Medtronic Inc, Minneapolis, MN, USA) while the 30-year-old only had implantation of a 28mm semi-rigid mitral ring annuloplasty (CG future^®^ 638R). Adequacy of the repair with only trivial regurgitation in both patients was confirmed by intra-operative trans-oesophageal echocardiography. Mitral valve repair in these patients with favourable mitral valve apparatus is recommended [11] as it precludes the adverse complications associated with prosthetic mechanical valves and anticoagulation including prosthetic valve thrombosis and embryotoxicity of warfarin [11–14].

The two patients were symptom-free at the 6-month follow-up clinic visit and trans-thoracic echocardiography done then did not show any deterioration in the degree of mitral valve regurgitation. Long term follow-up is being done on these patients. All cases were done using bicaval cannulation and antegrade cold blood cardioplegia and the aortic cross-clamp and cardiopulmonary bypass periods were noted to be slightly longer in patients with tetralogy of Fallot (TOF) compared with patients with non-cyanotic heart disease though the difference was not statistically significant. This is not unexpected since the complexity of repair in TOF is greater than for the non-cyanotic surgeries that were carried out in our series. Post-operatively, the patients were transferred to the intensive care unit and most had early extubation and weaning from mechanical ventilation after adequate recovery from anaesthesia using a fast-tracking approach which is recommended even in the paediatric population by various authors [15–17]. This approach resulted in over 75% of our patients being discharged from the hospital within 8 days of surgery (Table 3). Re-exploration for bleeding was carried out in 1 patient-a 20 year old man with classic tetralogy of Fallot. At re-exploration, diffuse oozing was seen from the pericardial and pleural edges and a few bleeding points along the right atriotomy suture line that required diathermic coagulation, use of surgicel^®^ (Ethicon Inc. San Lorenzo, Puerto Rico) and re-suturing over the suture line on the right atrium. Several researchers have previously discussed the defective coagulation in patients with cyanotic heart diseases, highlighting inferior clot formation occasioned by defects in both the intrinsic and extrinsic coagulation pathways [18–20]. This impairment in the clotting mechanism gets worse with time so that older patients with cyanotic heart diseases manifest worse clotting abilities than younger children hence the increased bleeding seen with this 20-year old man with tetralogy of Fallot.

## Conclusion

The safe conduct of open heart surgery is feasible in the completely private hospital setting in Nigeria with an initial blend of local and foreign teams which with time can lead to less dependency on foreign missions. The lessons from these will no doubt assist government-funded hospitals to stem the bureaucratic impediments that hinder the growth of this important contribution to heart care in the developing world.

### What is known about this topic

- In the West African sub-region, all open heart surgeries are carried out in government-funded hospitals;
- Inefficiencies and bureaucratic impediments hinder the growth of open heart surgery in Nigeria.

### What this study adds

- It is possible to perform open heart surgery in the completely privately funded hospital in West Africa;
- Repair of complex intra-cardiac defects are possible in the private hospital in West Africa with very low morbidity and mortality.

## Competing interests

The authors declare no competing interests.
